# The AGREE Enterprise: a decade of advancing clinical practice guidelines

**DOI:** 10.1186/s13012-014-0103-2

**Published:** 2014-08-15

**Authors:** Julie Makarski, Melissa C Brouwers

**Affiliations:** Department of Oncology, McMaster University, Juravinski Hospital and Cancer Centre Site, G Wing, Second Floor, Room 207, 1280 Main Street West Hamilton, Hamilton, Ontario Canada; Escarpment Cancer Research Institute, McMaster University, Hamilton, Ontario Canada; AGREE Enterprise Scientific Office, c/o McMaster University, Hamilton, Ontario Canada

**Keywords:** AGREE tools, AGREE II, AGREE Instrument, Clinical practice guidelines, Quality of care

## Abstract

**Background:**

The original AGREE (**A** ppraisal of **G** uidelines for **RE** search and **E** valuation) Instrument was published in 2003, and its revision, the AGREE II, in 2009. Together, they filled an important gap in the guideline and quality of care fields. Ten years later, the AGREE Enterprise reflects on a trajectory of projects and international collaboration that have contributed to advancing the science and quality of practice guidelines and the uptake of AGREE/AGREE II.

**Findings:**

The AGREE Enterprise has undertaken activities to improve the tool and to develop resources to support its use. Since 2003, the uptake and adoption of AGREE by the international community has been swift and broad. A total of 33 language translations of the original AGREE Instrument and the current AGREE II are available and were initiated by the international community. A recent scan of the published literature identified over 600 articles that referenced the AGREE tools. The AGREE tools have been widely received and applied, with several organizations having incorporated the AGREE as part of their formal practice guideline programs. Since its redevelopment in 2010, the AGREE Enterprise website (www.agreetrust.org) continues to experience steady increases in visitors per month and currently has over 10,000 registered users.

**Conclusions:**

The AGREE Enterprise has contributed to the advancements of guidelines through research activities and international participation by scientific and user communities. As we enter a new decade, we look forward to ongoing collaborations and contributing to further advancements to improve quality of care and health care systems.

In 2013, the AGREE (**A** ppraisal of **G** uidelines for **RE** search and **E** valuation) Enterprise celebrated its 10th year since the publication of the original AGREE Instrument [1]. Led by an evolving team of international researchers and clinical practice guideline (CPG) developers and users, the core goals of the AGREE program of research have remained the same: to improve the quality of patient care and health system performance through advances in the science and practice of CPGs by creating an appraisal tool capable of differentiating among CPGs of varying quality and to use the tool as a means to guide the development and reporting of CPGs. Using the 1997 *Cluzeau Instrument* as a springboard for its development, the original AGREE Instrument – a European-led and international effort - was first published in 2003 after the completion of a multi-year program of research by The AGREE Collaboration. The original AGREE Instrument’s uptake and adoption by the CPG community was swift and filled an important gap in the CPG and quality of care fields. The AGREE Instrument also contributed to the establishment of the Guidelines International Network (G-I-N) [2].

As with any appraisal instrument, there were opportunities to improve the usability, implementability and measurement properties of the original AGREE. This initiated another international effort, led by the AGREE Next Steps Research Consortium, and resulted in a revised tool, the AGREE II, published in 2009 [3]. The AGREE II is comprised of the appraisal tool (23 items, six domains, and a 7-point response scale) and User’s Manual, which supports its application and guides users on CPG development and reporting strategies. The work of the Consortium also produced the Global Rating Scale (GRS), a short and abridged version of the AGREE II that can be used for evaluation purposes when time and resources are limited [4].

The intent of the Consortium was not only to design a better quality tool, but also to create strategies to facilitate its use and to continue to raise the overall quality bar of the CPG enterprise internationally. To that end, three key implementation strategies resulted to achieve this goal. First, the AGREE Enterprise website [5] was launched in 2010. In addition to making available the original AGREE and the AGREE II instruments, their translations, and profiles of AGREE-related research projects, the website also included *“My AGREE”,* a platform that would enable online completion and storage of appraisals and the sharing of those appraisals among users.

Second, was the development of the AGREE II training tools*,* released in 2011. Through the AGREE A3 (Application, Appropriateness, Action) program of research, the team evaluated online tools via randomized-controlled trial to determine which strategy was most effective at promoting knowledge of the AGREE II and instilling confidence and satisfaction with its application [6]. The two online training strategies, an avatar-led tutorial and a dynamic training experience, in addition to the PDF version of AGREE II, are available through the AGREE website [7]. These training tools have been recently augmented by a comprehensive “Guidelines Resource Centre”, originally designed by the Capacity Enhancement Program of the Canadian Partnership Against Cancer [8]. It offers step-by-step training for how to create and implement high quality CPGs and includes practical resources, templates, videos, and a section outlining the AGREE II as a CPG development framework.

Third, and most recently in 2013, through an international collaboration of users, *“My AGREE PLUS”* was developed and launched. Building off the original *“My AGREE”* version, the *My AGREE PLUS* platform offers participants much greater functionality including the capacity to facilitate online completion and coordination of multiple-rater AGREE II assessments per CPG.

During the past 10 years, the AGREE instruments have been widely received and applied. As of 2013, there have been 33 translations of the original AGREE and the AGREE II and another 10 translations of the AGREE II are in development. We thank the international community for initiating and undertaking these translations. A recent initial scan of the published literature revealed over 600 articles that have referenced the AGREE tools. The AGREE II serves as a formal part of the CPG development or evaluation protocols of many established groups, including Cancer Care Ontario’s Program in Evidence-based Care (PEBC, Canada) [9], the National Institute for Health and Care Excellence (NICE, UK) [10], the World Health Organization (WHO) [11], and most recently, the National Academy of Clinical Biochemistry Laboratory Medicine (USA) [12]. The AGREE II has been used to inform the development of recently published CPG reporting standards including those developed by G-I-N [13] and the Institutes of Medicine’s Trustworthy Guidelines initiative [14]. The AGREE Enterprise website is visited and visited often – averaging 6,250 visitors per month. Further, there are over 10,000 *“My AGREE PLUS”* registered users (as of June 2014).

The AGREE II continues to be formally integrated in many graduate-level training programs including the Health Research Methodology graduate program at McMaster University (Canada); Dalhousie University’s Continuing Medical Education program (Canada); and the Department of Family Medicine at the University of Mississippi Medical Centre (USA). The topic of advancing the science of CPG appraisal contributed significantly to the training experiences of several international research leaders during their doctoral studies, including Francoise Cluzeau (UK: Cluzeau Instrument), Jako Burgers (Netherlands: original AGREE Instrument), and Michelle Kho (Canada: AGREE II instrument). Further, the AGREE II continues to be used in varied research and quality projects globally. For example, at the 2013 G-I-N Conference, 94 plenary, short presentations, and poster abstracts described using the instrument as part of, or as the object of, its research or project agenda [15].

Where to next? Although the work of the AGREE Enterprise is ongoing, its focus has changed direction. At this time, no additional work is planned by the team to modify or continue to refine the AGREE II, although the team collaborates with other groups (e.g. the National Guidelines Clearinghouse) to support the adaption of the instrument to meet the needs of the users. There are clearly items in which methodologies and gold standard criteria for performance are required (e.g. editorial independence); we encourage the research community to take this on.

Our team is now focused on the development of a complementary tool to the AGREE II, the AGREE REX (**R** ecommendation **EX** cellence). Whereas the AGREE II targeted the methodological issues for developing the whole guideline report, the team wishes to concentrate its efforts on a program of research aimed at optimizing the implementability, applicability, and quality of the CPG recommendations. As a first step to this goal, the team collaborated widely with other research groups to create the GUIDE-M (**GU** ideline Implementability for **D** ecision **E** xcellence **M** odel); a comprehensive evidence-informed framework of intrinsic CPG factors associated with the uptake of recommendations. In addition, the GUIDE-M allows us to plot existing CPG tools and advancements to identify (i) where sufficient gains have been made and as such, where no additional action may be required and (ii) gaps where investment of research efforts may be most beneficial. GUIDE-M is undergoing its final consultation phase and will be publicly available in 2014. The GUIDE-M will serve as the foundation from which the AGREE-REX will be designed. The team is also currently leading a new project to create a tool to facilitate the appraisal, development and reporting of health systems guidance.

Over the past 10 years the AGREE Enterprise has contributed to the advancements of CPGs through its various research activities and international participation by the scientific and user communities. As we enter a new decade, we look forward to ongoing and expansive collaborations and contributing to further advancements that will be useful to improve quality of care and health care systems.

## References

[1] Cluzeau F, Burgers J, Brouwers M, Grol R, Makela M, Littlejohns P, Grimshaw J, Hunt C (2003). Development and validation of an international appraisal instrument for assessing the quality of clinical practice guidelines: the AGREE project. Quality & Safety in Healthcare.

[2] Guidelines International Network: Guidelines International Network., [www.g-i-n.net]

[3] Brouwers M, Kho ME, Browman GP, Cluzeau F, Feder G, Fervers B, Hanna S, Makarski J (2010). AGREE II: advancing guideline development, reporting and evaluation in healthcare. Can Med Assoc J.

[4] Brouwers MC, Kho ME, Browman GP, Burgers J, Cluzeau F, Feder G, Fervers B, Graham I, Grimshaw J, Hanna S, Littlejohns P, Makarski J, Zitzelsberger L (2012). The global rating scale complements the AGREE II in advancing the quality of practice guidelines. J Clin Epidemiol.

[5] AGREE Enterprise: AGREE enterprise website., [www.agreetrust.org]

[6] Brouwers MC, Makarski J, Durocher L, Levinson A (2011). E-learning interventions are comparable to user’s manual in a randomized trial of training strategies for the AGREE II. Implement Sci.

[7] AGREE Enterprise: AGREE enterprise website – AGREE II training tools., [http://www.agreetrust.org/resource-centre/agree-ii-training-tools/]

[8] Canadian Partnership Against Cancer Capacity Enhancement Program: Cancerview –guidelines resource centre., [http://www.cancerview.ca/cv/portal/Home/TreatmentAndSupport/TSProfessionals/ClinicalGuidelines/GRCMain?lang=en]

[9] Cancer Care Ontario: Cancer Care Ontario – program in evidence-based care., [https://www.cancercare.on.ca/about/programs/pebc/]

[10] National Institute for Health and Care Excellence: National Institute for Health and Care Excellence., [http://www.nice.org.uk]

[11] World Health Organization: WHO Handbook for Guideline Development. 2012

[12] National Academy of Clinical Biochemistry Laboratory Medicine: American Association for Clinical Chemistry., [www.aacc.org]

[13] Qaseem A, Forland F, Macbeth F, Ollenschläger G, Phillips S, Van der Wees P (2012). Guidelines International Network: towards International Standards for Clinical Practice Guidelines. Ann Intern Med.

[14] Graham R, Mancher M, Wolman DM, Greenfield S, Steinberg E (2011). Committee on Standards for Developing Trustworthy Clinical Practice Guidelines, Board on Health Care Services: Clinical Practice Guidelines We Can Trust.

[15] G-I-N Conference San Francisco 2013: Conference proceedings. 2013, 22(1). Available at: [http://qualitysafety.bmj.com/content/22/Suppl_1.toc].

